# Predicting Adolescent Arithmetic and Reading Dysfluency

**DOI:** 10.1177/00222194241275644

**Published:** 2024-09-24

**Authors:** Tuire Koponen, Kenneth Eklund, Kaisa Aunola, Anna-Maija Poikkeus, Marja-Kristiina Lerkkanen, Minna Torppa

**Affiliations:** 1 University of Jyväskylä, Finland

**Keywords:** arithmetic fluency, reading fluency, learning difficulties, longitudinal prediction

## Abstract

The long-term negative consequences of learning difficulties have been acknowledged. Nonetheless, research is still scarce regarding the prediction of adolescent difficulties in reading and arithmetic skills. The present study examines at which age phase and with what kind of constellation of parent- and child-related factors can adolescent difficulties in arithmetic and/or reading fluency be successfully predicted. A sample of Finnish children (*N* = 941) was followed from the onset of kindergarten (at age 6) through adolescence (ages 13–16). Children’s cognitive skills were assessed in kindergarten, and arithmetic and reading fluency were examined in Grades 2, 4, 6, 7, and 9. Parents’ self-report data were collected on their own learning difficulties and educational level. Scoring below the 16th percentile in both Grades 7 and 9 was set as the criterion for dysfluency either in reading (*n* = 87, 9.2%) or arithmetic (*n* = 84, 8.9%). Adolescent dysfluency in both domains was moderately predicted by parental measures and kindergarten cognitive skills. Although adding school-age fluency measures clearly increased both the predictability and specificity of models up to Grade 4 for both skills, knowledge of letters’ names, counting, and visuospatial skills remained unique predictors of dysfluency in adolescence.

Learning difficulties in reading and mathematics can have serious negative impacts on school achievement and successful educational attainment (e.g., Hakkarainen et al., 2016), constituting a risk factor for adolescents’ and adults’ well-being and mental health (e.g., Willcutt et al., 2019). Importantly, reading difficulties (RD) and mathematics difficulties (MD) tend to persist into adulthood, especially if students do not receive effective interventions and intensified support at school (Kaufmann et al., 2011; Wilson et al., 2015). Evidence has accumulated on the predictors of learning difficulties concerning the early grades. Nonetheless, research is still scarce regarding the prediction of adolescent learning difficulties (e.g., Nelson & Powell, 2018). The studies that identify predictors for early-emerging learning difficulties may not apply to all adolescents, as previous studies have suggested that, at least in reading, both late-emerging and resolving learning difficulty profiles exist (e.g., Torppa et al., 2015). Different developmental profiles might relate to varying phases of skill development (e.g., learning of decoding/accurate calculation skills vs. gaining fluency in reading/arithmetic), which are likely to have partially different requirements and underlying cognitive processes.

Previous studies of learning difficulties have mostly focused on the primary school years (Grades 1 to 6 corresponding ages from 7 to 12 years in Finland; ages from 5 to 11 in Britain), that is, the basic skill acquisition phase, when accurate decoding and early computational skills constitute central educational aims. Due to the scarcity of long-term longitudinal studies on this subject, knowledge is lacking regarding the age phases, skills, and background factors that provide reliable predictions of reading or mathematics difficulties in adolescence, manifested as dysfluency. A critical question is whether we can reliably predict dysfluency statuses in adolescence only after children have received several years of formal instruction and typically have made the developmental transition from one-by-one coding to the more automatic, retrieval-based phase in reading (Ehri & Robbins, 1992) and arithmetic (Imbo & Vandierendonck, 2005). Alternatively, is knowledge of the earlier academic skill acquisition phase already informative of skills in adolescence? It is relevant to early effective interventions if we can predict adolescent difficulties at a younger age, perhaps before children enter school, using information about the early cognitive skills underlying the development of arithmetic and reading skills or information on parents’ learning difficulties. Information that guides and improves the reliable prediction of children who need intensified support in adolescence is important and critical for allocating educational support resources wisely.

## Dysfluency as a Hallmark in Reading and Mathematical Difficulties

In the context of academic skills, *fluency* refers to the ability to perform a specific task or skill smoothly and effortlessly, with a high degree of proficiency. In the present study, the term *fluency* refers to the ability to read or calculate with speed and accuracy (see Binder, 1996). The typical manifestation of reading and mathematics difficulties after the foundational skill acquisition phase is dysfluency, manifested as deficits in smooth word identification in reading (Wimmer & Mayringer, 2002) and in arithmetic fact retrieval and the use of efficient procedural strategies in mathematics (Geary, 1993). Learning foundational decoding and arithmetic calculation skills is a key focus of formal instruction during the first years of primary school. Despite the repeated practice provided by the school’s instruction, a subgroup of adolescents (e.g., Eklund et al., 2015; Torppa et al., 2015) and adults (Leinonen et al., 2001) experience difficulties in developing automaticity in decoding, and dyslexia is prevalent even in the most transparent orthographies, such as Finnish. Automatization difficulties can similarly be found in arithmetic. In the age-typical development of arithmetic skills, children usually start using fact retrieval as the main strategy for solving arithmetic problems around age 9 (Imbo & Vandierendonck, 2005). Adults with mathematics difficulties show fact retrieval deficits and must use counting-based backup strategies to solve even simple arithmetic problems (Kaufmann et al., 2011). In addition to retrieval deficits, difficulties in arithmetic fluency can arise from a lack of understanding of arithmetic concepts or problems with procedural skills in executing step-by-step procedures or algorithms correctly and efficiently (Geary, 1993). Difficulties may involve applying the appropriate rules and operations to manipulate numbers and solve arithmetic problems accurately. Dysfluency has a large impact on later learning in both main academic domains. The automaticity of decoding releases the capacity for reading comprehension. In addition, fluent calculation skills release the cognitive capacity for arithmetic problem-solving and are related to overall math proficiency (Nelson & Powell, 2018). Moreover, both skills are needed for learning other academic subjects.

## Family Risk as a Predictor of Later Reading and Mathematics Difficulties

The earliest possible time to predict later learning difficulties is before the child is born, using information regarding parents’ learning difficulties. Parental learning difficulties are known to constitute a familial risk factor for children in the domains of reading (e.g., Puolakanaho et al., 2007; Snowling & Melby-Lervåg, 2016) and mathematics (e.g., Shalev et al., 2001). The hereditary effect of a parent’s reading difficulties (RD) on a child’s reading skills is quite substantial. However, the prevalence of dyslexia among children with a parent with dyslexia is estimated to vary greatly (29%–66%) depending on the sample and assessment method of parental skills (Snowling & Melby-Lervåg, 2016). Significantly less is known about family risk (FR) for mathematics difficulties. Nevertheless, it is suggested that, as with other specific learning difficulties, math difficulties are characterized by significant family aggregation. A total of 66% of mothers, 40% of fathers, 53% of siblings, and 44% of second-degree relatives have been reported to have learning difficulties in mathematics (Shalev et al., 2001). More longitudinal studies are still needed to clarify the role of FR as a long-term predictor of adolescent fluency problems, among other parental factors. The parental risk in the present study refers to parents’ self-reported difficulties in reading or math.

Parents’ educational level, among other socioeconomic factors, has been found to predict a relatively small proportion of the variance in children’s academic achievement among the Finnish population (e.g., Marks, 2006). Parental education has not been found to have predictive value for reading and arithmetic dysfluency among Finnish third graders (Pulkkinen et al., 2022). However, in a previous longitudinal study using the same data from Grades 1 to 7 with a correlative approach (Korpipää et al., 2017), it was documented that parental education predicted the shared performance level in reading and arithmetic in adolescence, although no significant association was found at the beginning of schooling.

## Kindergarten Skills as Predictors of Adolescent Reading and Arithmetic Dysfluency

Previous research on reading and arithmetic fluency in primary school years has provided important knowledge of the core precursors of learning difficulties, such as number sense (mapping number symbols with quantities, counting, etc.) in arithmetic (e.g., Kroesbergen et al., 2023) and phonological awareness (PA), rapid automatized naming (RAN), and letter-sound knowledge (LK) in reading (e.g., Manis et al., 2000; Puolakanaho et al., 2007). Although numerous studies have examined the precursors of difficulties manifested in the early grades, only a handful of studies have investigated early cognitive predictors of adolescent learning difficulties. The studies suggest that RAN continues to be an important early predictor of adolescent reading, while the effects of letter knowledge (LK) and PA have been documented inconsistently (e.g., Landerl & Wimmer, 2008; Torppa et al., 2015). Psyridou et al. (2022) recently examined kindergarten predictors of reading dysfluency in the present sample when comparing three statistical models based on their accuracy in predicting RD. In all models, RAN and LK were significant cognitive predictors of RD when reading fluency in primary school was not considered.

In the few existing studies in mathematics research, kindergarten-age number concept skills and verbal counting (Koponen et al., 2019) have been shown to predict seventh graders’ mathematics difficulties. However, due to the limited number of longitudinal studies focusing on predicting learning difficulties after primary school, the early predictors of dysfluency status in adolescence remain largely unexplored. Accordingly, further studies are necessary with appropriate sample sizes. In particular, there is a lack of research exploring the accuracy of the prediction at different time points, that is, examining when adolescent dysfluency can be accurately predicted. In addition to numerical skills, research suggests that visuospatial skills are warranted to take into account. Particularly, the early ability to detect spatial relations is documented to significantly contribute to later math achievement and arithmetical difficulties (Wang et al., 2021; Zhang et al., 2020). This contribution remains significant even when considering other visuospatial skills, such as mental rotation and spatial perception (Wang et al., 2021).

## Primary School Arithmetic and Reading Skills as Predictors of Difficulties

When predicting adolescent reading and arithmetic dysfluency status, the strongest expected predictors are the same skills at earlier grade levels. Previous longitudinal studies have not directly examined the extent to which fluency in primary school years predicts reading and arithmetic dysfluency status in adolescence, although the persistence of dysfluency status in reading has been the focus of several studies. Landerl and Wimmer (2008) showed that 70% of the observed dysfluent readers in Grade 1 were still poor readers in Grade 8. Unstable reading fluency was reported in a study of the present sample across Grades 2 to 6 (Psyridou et al., 2020) and in another Finnish sample following children across Grades 2 to 8 (Torppa et al., 2015). Although the difficulty persists for many children, for some individuals, RDs sometimes emerge only in later grades. For some, the difficulties are resolved by adolescence.

There is limited knowledge regarding the persistence of mathematics difficulties from the beginning of schooling to adolescence. However, in a study of children diagnosed with dyscalculia in Grade 5, Shalev et al. (2005) found that 95% still performed poorly in arithmetic, scoring within the lowest quartile in Grade 11. In an analysis of the present sample across Grades 1 to 4, the difficulties in arithmetic fluency identified at the end of Grade 1 did not exhibit strong stability during the follow-up by Grade 4 (Koponen et al., 2018). As changes in the fluency difficulty status are identified, it is important to examine to what extent the prediction of adolescent dysfluency can be improved by utilizing information on reading and arithmetic fluency across the grades of primary school.

## The Present Study

This study aims to fill the gap related to the early prediction of adolescent reading and arithmetic dysfluency status. The goal of the present study is to analyze the unique, cumulative value of information related to cognitive skills in kindergarten and the development of reading and arithmetic skills across the primary school years. Of specific interest is whether kindergarten skills predict dysfluency over the level of reading and arithmetic fluency at school age. In addition, to what extent does reading or arithmetic performance in later grades predict future difficulties in adolescence?

*Dysfluency* is defined as a low ability to read or calculate with speed and accuracy. To increase the reliability of the prediction of participants with dysfluency in adolescence, a double-occurrence criterion was adopted (dysfluency identified at Grades 7 and 9, 13–16 years). The design involved an examination of the unique and cumulative values of information related to the family’s risk for learning difficulties (FR), parents’ level of education, children’s cognitive skills in kindergarten, and their reading and arithmetic skills after the basic skill acquisition phase in formal education (Grade 2) and in later grades (Grades 4 and 6), when both skills become more automatized.

The present study addresses the following research question:

What is the role of (a) FR, (b) parental education, (c) kindergarten cognitive skills (i.e., LK, PA, RAN, counting, spatial relations, and number concepts), (d) school-age reading, and (e) arithmetic fluency in predicting the reading and/or arithmetic dysfluency status in adolescence?

We are, first, interested in finding out not only the extent to which FR, parental education, and kindergarten cognitive skills can predict dysfluency status in adolescence, but also which predictors uniquely contribute when inspected simultaneously in the same model and whether these contributions remain after the inclusion of school-age reading and/or arithmetic fluency skills. Second, we are interested in determining how well and how early we can predict adolescents’ dysfluency status. Therefore, we examine whether and to what extent the prediction of the kindergarten models improves after the inclusion of reading and/or arithmetic fluency measures (a) after the basic skill acquisition phase (Grade 2 spring, 8–9 years) and (b) during the fluency-building phase (Grade 4 spring, 10–11 years, and Grade 6 spring, 12–13 years). Finally, this work tested the ability of reading and/or arithmetic fluency measures separately in each grade (and without parental and kindergarten measures) to predict dysfluency statuses in adolescence.

## Method

This study is part of an extensive longitudinal study conducted in Finland (Lerkkanen, Niemi, et al., 2006), in which 1,880 children and their classmates were followed from kindergarten to Grade 9. The sample was recruited from four municipalities from central, western, and eastern Finland. Written parental consent was obtained from all participants. The sample represented the average family’s background characteristics in Finland (Statistics Finland, 2007).

### Participants

The individuals in this study were children with full data on all predictors and outcome measures: thus, 941 children (46.4% girls, age at kindergarten *M* = 6.26 years, *SD* = 0.29 years). Little’s MCAR test indicated that missingness was not completely random, χ^2^(*df* = 313) = 491.88, *p* < .001. Those included in this study had better skills in all measures used. However, there was no difference in the number of FR children or in parental education compared with the full sample. Effect sizes (Cohen’s *d*) between those with and without missing data were, however, negligible or small (0.05–0.37; Cohen, 2016). The vast majority (71%) of children originated from nuclear families, 17.3% from single-parent families, and 11.7% from blended families or from families in which the parents were divorced and the child had two homes. A total of 31.5% of the children’s mothers had a master’s degree or higher, 34.5% had a bachelor’s degree or vocational college degree, 27.3% had a vocational school degree, and 6.7% had no education beyond comprehensive school. The subjects were all native Finnish speakers with no documented intellectual or sensory deficits.

### Measures

**Figure 1.**
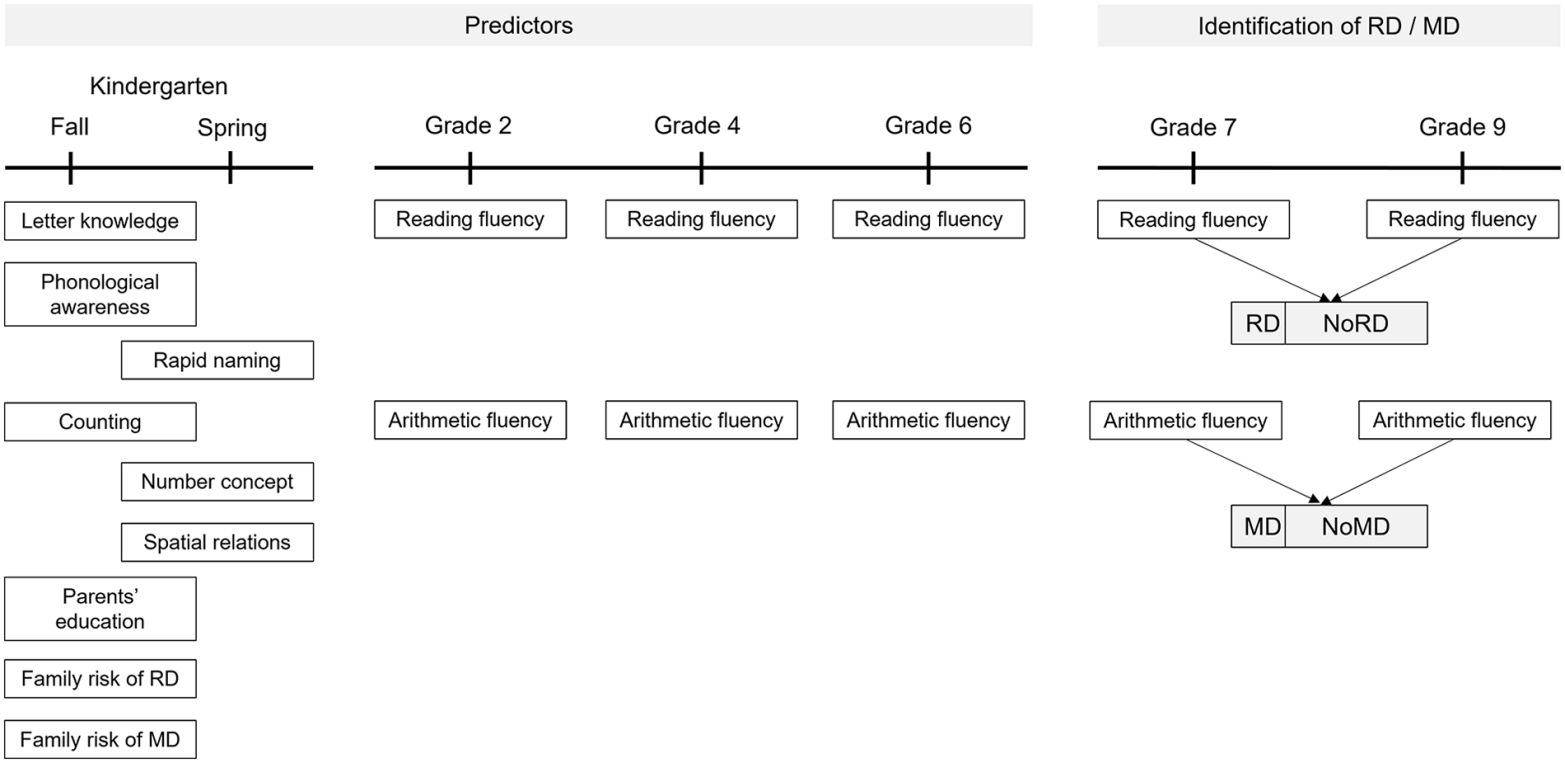
Measures at Different Assessment Points Used as Predictors in Regression Models and in Identification of RD and MD Status in Adolescence. *Note*. RD = reading difficulties; MD = mathematics difficulties.

In the present study, we used data from assessments conducted in kindergarten and at schools in Grades 2, 4, 6, 7, and 9 during the regular day (for the design, see Figure 1). Tasks were administered by trained testers (researchers or research assistants who were students of psychology or education). Kindergarten skills were evaluated in individual situations, and school-age arithmetic and reading skills were examined in group situations. The measures are described briefly below, and a more detailed description can be found in supplemental materials (see Supplemental Table S1).

#### Parental Measures

Parental education as well as reading and mathematics difficulties were investigated with a questionnaire issued when children were in kindergarten. Parents rated their education on a 7-point scale from 1 (*no vocational education*) to 7 (*licentiate or doctoral degree*). The sum score was computed as an average of both parents’ individual scores. Parents rated their experiences with learning difficulties in reading and/or mathematics on a 3-point scale (1 = *no difficulties*, 2 = *some difficulties*, 3 = *clear difficulties*). To account for individual differences in interpreting the term “difficulties,” we included both “clear difficulties” and “some difficulties” options on the scale to clearly separate those with no difficulties. A dichotomic FR variable was recoded for both reading (FR_RD_) and mathematics (FR_MD_). An FR was defined if the mother or father reported that they had experienced some or clear learning challenges.

#### Kindergarten Cognitive Skills

The LK task required children to name-read a list of the 29 letters of the Scandinavian variant of the Latin alphabet used in Finnish. The sum score of correctly named letters was used as a variable. Cronbach’s alpha was .95. Phonological awareness was assessed using the initial phoneme identification test from ARMI test material (Luku- ja kirjoitustaidon arviointimateriaali 1. luokalle; Lerkkanen, Poikkeus, et al., 2006). The child was asked to select the correct picture of the four options based on the oral presentation of the initial phoneme relating to one target. The sum score of correct answers was used as a variable. Cronbach’s alpha was .75.

The rapid naming (RAN) of objects was assessed using the standard procedure (see Denckla & Rudel, 1974), in which the child was asked to name as rapidly as possible a series of visual stimuli. Five familiar objects (pictures of fish, house, ball, car, pencil), which all represent words composed of two syllables in Finnish, were arranged in a 5 × 10 matrix in a random order where each object picture is replicated 10 times. The total matrix completion time was used as a variable.

Counting was assessed using the Number Sequences Test (Salonen et al., 1994), in which the child was first asked to recite number words forward from 1 to 31 and from 6 to 13, tapping into the knowledge of the correct sequence of number words and the ability to start from the middle of the sequence, requiring automatic access to sequence knowledge without starting from the beginning with the number one. Backward item covered counting from 12 to 7 and from 23 to 1. Moreover, 2 points were awarded for the correct outcome, 1 point for completing the task with up to two errors, and 0 points if the child made more than two errors or failed to complete the task. The sum score of points was used as a variable. Cronbach’s alpha was .63. The number concept was assessed using the School Entry Screener (Elomäki et al., 1999), covering cardinal (e.g., draw as many/one more/one less) and ordinal number knowledge (e.g., mark the first/fourth/seventh ball) and basic mathematical concepts. The sum score of correct answers was used as a variable. Cronbach’s alpha was .62. Visuospatial skills were gauged using a time-limited subtest of spatial relationships from the Woodcock and Johnson (1977) test battery. The children were instructed to identify the subset of pieces needed to form a complete shape. The sum score of correct answers was used as a variable.

#### Arithmetic Fluency

Arithmetic fluency was assessed using two group-administered and time-limited paper-and-pencil tests:

1. An arithmetic test with a time limit of 3 min (Aunola & Räsänen, 2007) containing 28 items covering all four arithmetic operations in single-digit, multidigit, and decimal numbers. The difficulty of tasks increased over the years, and the easiest items were replaced with more challenging ones to align with curriculum changes across the grades. In Grade 1 to 3, test included 14 addition items (e.g., 2 + 1 =, 3 + 4 + 6 =), and 14 subtraction items (e.g., 4 – 1 =, 20 – 2 – 4 =). In Grade 4, six new and more difficult items of addition, subtraction, multiplication (e.g., 12 × 28 =), division (e.g., 240 ÷ 80 =), or mixed mode calculation (e.g., 40 ÷ 8 – 3 =) were added and the six easiest items (e.g., 4 – 1 =, 2 + 1 =) were removed. In Grade 6, three new and more difficult items of addition, subtraction (e.g., 84 + 13 – 27 =), division (e.g., 57 ÷ 5 =), or calculation with decimals (e.g., 106.2 – 30.04 =) were added to and the three easiest items (e.g., 8 + 6 =, 9 + 3 =) were removed. In Grade 7, three new and more difficult items of mixed mode calculations (e.g., 40 ÷ 8 – 3 = x, 6 × 4 + 1 = x – 21), or calculation with decimals (e.g., 28.3 + 19.8 = x) were added and the three easiest items were removed. No changes were made in Grade 9.
2. A multiplication fluency test with a 2-min time limit (Koponen & Mononen, 2010) containing 120 items with a multiplicand and multiplier less than 10. The mean value of the standardized variables was used as the outcome variable for arithmetic fluency. The only exception was second grade, when only arithmetic fluency was tested because all multiplication tables were not taught yet. Cronbach’s alphas for Grades 4 and 6 were .70 and .74. For Grades 7 and 9, they were .74 and .75, respectively.

#### Reading Fluency

Reading fluency was assessed using two group-administered and time-limited silent reading tests. Both reading fluency tasks are standardized tests and part of national batteries, which are routinely used in Finnish schools to assess students’ ability to decode words fast and accurately. In the ALLU test (ALLU–Ala-asteen lukutesti [ALLU–Reading Test for Primary School]; Lindeman, 2000), the child matched one of four phonologically similar words with a picture within a 2-min time limit. In the word chain task (Nevala & Lyytinen, 2000), 10 items composed of chains of four to six words without any space between the words were presented as rows on a sheet of paper. Children were asked to draw a boundary line between the consecutive words. Time limits varied according to grade (from 1 min to 1 min 25 s). The mean value of the standardized variables was used as the outcome variable for reading fluency. The Cronbach’s alphas for Grades 2, 4, and 6 were .63, .69, and .66, and for Grades 7 and 9, they were .76 and .79, respectively. Among a subsample of the participants (*N* = 265), the correlation between the silent reading fluency measure and the individually assessed fluency of reading aloud varied from .67 in Grade 7 to .64 in Grade 9.

### Data Analysis

The commonly used cut-off criterion, one standard deviation below the mean (lowest 16th percentile), was applied to define difficulties (e.g., Geary et al., 2007; Landerl et al., 2009). Children were classified as having difficulties in reading (RD) or mathematics (MD) in adolescence if they scored below the 16th percentile (–1 *SD*) in the reading or arithmetic fluency tasks in Grades 7 and 9. The 16th percentile was calculated for all participants attending Grade 7 (*N* = 1,763) or Grade 9 (*N =* 1,707) assessments. In addition, 87 adolescents (9.2%) fulfilled the reading criterion, and 84 (8.9%) fulfilled the arithmetic criterion.

To examine differences between groups with and without dysfluency (RD vs. NoRD and MD vs. NoMD), cross-tabulation and chi-square tests were used with categorical variables (gender, FR for reading and mathematics difficulties) and independent-samples *t* tests with continuous variables (parental education, cognitive skills in kindergarten, and school-age reading and arithmetic fluency in Grades 2, 4, and 6). In addition, a nonparametric Mann–Whitney *U* test was used due to slightly skewed distributions in the LK, PA, RAN, counting, and number concepts. However, due to the similarity of the results with those of the *t* tests, the parametric *t* tests are reported except for the number concept, in which the results of both tests are reported. Due to 28 paired comparisons, commonly used *p* values were divided by 28 to avoid Type II errors. Therefore, * *p* < .002, was used instead of *p < .05, **p < .01, and ***p < .001. To answer RQ1, hierarchical binary logistic regression analyses were performed separately for reading dysfluency and arithmetic dysfluency status. In Model 1, FR and parental education were entered in the first step, followed by all kindergarten cognitive skill measures in the second step. Models 2 to 4 were run to examine whether school-age fluency measures exhibited additive predictive power for dysfluency statuses in adolescence. In these models, the significant predictors of Model 1 were entered as the first step, and in Grades 2, 4, and 6, the respective fluency measure (reading/arithmetic) was entered in the second, third, and fourth steps, respectively. Standardized measures of continuous predictors were used in all three models to increase the comparability between values related to different predictors. Receiver operating characteristic (ROC) analysis was performed separately for each model, and the area under the ROC curve (AUC) as an index of overall location of the ROC curve as well as sensitivity and specificity values were inspected. *Sensitivity* refers to the model’s ability to correctly predict individuals with difficulty in adolescence, and *specificity* refers to the ability to correctly predict individuals without difficulty in adolescence. When using continuous predictors, the sensitivity and specificity depend on the cut-offs used. The ROC curve is a visual representation describing the model’s ability to predict individuals with different outcome statuses using varying cut-offs. Moreover, the AUC value represents the degree to which the model distinguishes between two classes, which enables comparisons between models (Fawcett, 2006).

## Results

### Predicting RD Status in Adolescence

Both FR_RD_ and FR_MD_ were at least twice as common in the RD group compared with the NoRD group (see Table 1). Parental education did not differ between the RD and NoRD groups. However, all the cognitive skills except the number concept were lower in the RD group compared with the NoRD group. Moreover, the NoRD group outperformed the RD group in their levels of reading and arithmetic fluency throughout all school-age assessments. Effect sizes varied from moderate to large (see Table 2).

**Table 1.** Percentages of Girls and Children With FRRD and FRMD.

	RD vs. NoRD			MD vs. NoMD		
Group	RD, *N* = 87	NoRD, *N* = 854	χ^2^(1)	MD, *N* = 84	NoMD, *N* = 857	χ^2^(1)
Girls	21.8%	48.9%	23.32***	52.4%	45.9%	1.31
FR_RD_	19.5%	7.7%	13.70***	11.9%	8.5%	1.09
FR_MD_	13.8%	6.0%	7.73**	11.9%	6.2%	4.01*

*Note.* FR_RD_ = family risk for reading difficulties; FR_MD_ = family risk for mathematics difficulties; RD = reading difficulties; MD = mathematics difficulties; NoRD/NoMD = no difficulties in corresponding domain.

* *p* < .05. ** *p* < .01. *** *p* < .001.

**Table 2.** Descriptive Statistics and Group Comparisons of Standardized Measures.

	RD vs. NoRD						MD vs. NoMD					
	RD (*N* = 87)		NoRD (*N* = 854)				MD (*N* = 84)		NoMD (*N* = 857)			
Measures	*M*	*SD*	*M*	*SD*	*t*(939)	ES^a^	*M*	*SD*	*M*	*SD*	*t*(939)	ES^a^
Parent’s education	–0.27	0.99	0.03	1.00	2.66	0.30	–0.33	0.97	0.03	1.00	3.18*	0.36
Letter knowledge	–0.78	0.94	0.08	0.97	7.93*	0.89	–0.72	0.94	0.07	0.98	7.13*	0.81
Phonological awareness	–0.50	0.98	0.05	0.98	5.00*	056	–0.44	1.01	0.04	0.99	4.28*	0.48
Rapid automatized naming	–0.62	0.94	0.06	0.98	6.41*	0.70	–0.41	0.90	0.04	1.00	3.95*	0.45
Counting	–060	0.98	0.06	0.98	5.94*	0.67	–0.89	0.89	0.09	0.97	8.88*	1.02
Spatial relations	–0.36	0.92	0.04	1.00	3.56*	0.40	–0.60	0.87	0.06	0.99	5.84*	0.67
Number concept	–0.22	1.13	0.02	0.98	1.95	0.24	–0.41	1.18	0.04	0.97	4.00*	0.45
Gr. 2 reading fluency	–1.06	0.61	0.11	0.97	15.94*	1.24	–0.60	0.95	0.06	0.99	5.87*	0.67
Gr. 2 arithmetic fluency	–0.64	0.95	0.07	0.98	6.39*	0.73	–1.19	0.81	0.12	0.94	13.92*	1.41
Gr. 4 reading fluency	–1.24	0.67	0.13	0.94	17.34*	1.49	–0.58	1.03	0.06	0.98	5.67*	0.65
Gr. 4 arithmetic fluency	–0.56	1.01	0.24	0.90	7.88*	0.88	–1.12	0.88	0.29	0.84	14.56*	1.67
Gr. 6 reading fluency	–0.99	0.65	0.23	0.78	16.18*	1.56	–0.41	0.89	0.17	0.82	5.96*	0.70
Gr. 6 arithmetic fluency	–0.62	0.65	0.16	0.86	10.07*	0.93	–1.04	0.58	0.19	0.81	17.53*	1.55

*Note.* Effect sizes (ES) were estimated with Cohen’s *d* (computed with pooled standard deviations, considering the number of participants in each group). RD = reading difficulties; MD = mathematics difficulties; NoRD/NoMD = no difficulties in corresponding domain.

* *p* < .002.

Spearman correlations between the predictors and the outcomes are presented in supplemental materials (see Supplemental Table S2). In the hierarchical binary logistic regression model (Model 1), which predicts adolescent reading dysfluency status through parental predictors, FR_RD_ was the only significant parental predictor, explaining 4% of the variance (see Table 3). It remained important even after adding cognitive skills in Step 2. Of the cognitive skills, LK and RAN were marked predictors of the adolescents’ reading dysfluency status. The model explained 18% of the variance, and the AUC value of 0.75 suggested that the model’s ability to predict RD statuses in adolescence was moderate. Using a cutoff probability that resulted in 90.8% sensitivity in binary logistic regression, the model correctly predicted adolescents’ reading disability status in only half of the cases due to low specificity (46.3%) (see Table 4). As there was also a significant disparity between the RD and NoRD groups in familial risk for mathematical difficulties (FR_MD_), we further investigated the predictive role of FR_MD_ together with FR_RD_ in determining dysfluency statuses in reading. However, our analysis indicated that FR_MD_ did not emerge as a significant predictor of dysfluency beyond the effects of FR_RD_.

**Table 3.** Predicting Reading Difficulties (RD) Status in Adolescence.

Predictors	Time points of the predictors included in the model											
	Kindergarten			Kindergarten and Grade 2			Kindergarten, Grades 2 and 4			Pre-school, Grades 2, 4, and 6		
	β	*SE*	Odds ratio	β	*SE*	Odds ratio	β	*SE*	Odds ratio	β	*SE*	Odds ratio
	Step 1			Step 1			Step 1			Step 1		
FR of RD	0.77*	.33	2.16	0.70*	.36	2.02	0.75	.39	2.11	0.81*	.41	2.25
Parents’ education	–0.04	.12	0.96	—	—	—	—	—	—	—	—	—
	Step 2											
LK	–0.62***	.16	0.54	–0.56***	.14	0.57	–0.48**	.15	0.62	–0.45**	.16	0.64
PA	–0.04	.13	0.96	—	—	—	—	—	—	—	—	—
RAN	0.38***	.11	1.46	0.19	.12	1.21	0.15	.13	1.16	0.13	.14	1.14
Counting	–0.14	.14	0.87	—	—	—	—	—	—	—	—	—
Spatial relations	–0.21	.13	0.81	—	—	—	—	—	—	—	—	—
Number concept	0.06	.12	1.06	—	—	—	—	—	—	—	—	—
				Step 2			Step 2			Step 2		
Gr. 2 reading fluency	—	—	—	–1.79***	.22	0.17	–0.93***	.25	0.39	–0.77**	.26	0.46
							Step 3			Step 3		
Gr. 4 reading fluency	—	—	—	—	—	—	–1.65***	.25	0.19	–0.94**	.29	0.39
										Step 4		
Gr. 6 reading fluency	—	—	—	—	—	—	—	—	—	–1.29***	.25	0.28
	Nagelkerke *R*^2^ = .18***			Nagelkerke *R*^2^ = .36***			Nagelkerke *R*^2^ = .47***			Nagelkerke *R*^2^ = 52***		
AUC	.75***^a^			.89***			.91***			.93***		

*Note.* Standardized scores of continuous predictors were used in the model to increase comparability of the odds ratios related to different predictors. The beta-coefficients related to the final model after all steps in each model are reported in the table. LK = letter knowledge; RAN = rapid automatized naming; PA = phonological awareness.

a Area under the ROC curve calculated using only the significant predictors of RD status in adolescence.

* *p* < .05. ** *p* < .01. *** *p* < .001.

**Table 4.** Predicting Mathematics Difficulties (MD) Status in Adolescence.

Predictors	Time points of the predictors included in the model											
	Kindergarten			Kindergarten and Grade 2			Kindergarten, Grades 2 and 4			Pre-school, Grades 2, 4, and 6		
	β	*SE*	Odds ratio	β	*SE*	Odds ratio	β	*SE*	Odds ratio	β	*SE*	Odds ratio
	Step 1			Step 1			Step 1			Step 1		
FR of MD	0.52	.41	1.69	—	—	—	—	—	—	—	—	—
Parents’ education	–0.12	.13	0.89	—	—	—	—	—	—	—	—	—
	Step 2											
LK	–0.29	.16	0.75	—	—	—	—	—	—	—	—	—
PA	0.07	.13	1.07	—	—	—	—	—	—	—	—	—
RAN	0.13	.12	1.13	—	—	—	—	—	—	—	—	—
Counting	–0.68***	.15	0.51	–0.47**	.15	0.63	–0.31*	.15	.73	–0.26	.16	.77
Spatial relations	–0.46***	.13	0.63	–0.36**	.13	0.70	–0.29*	.14	.75	–0.23	.15	.79
Number concept	–0.08	.11	0.93	—	—	—	—	—	—	—	—	—
				Step 2			Step 2			Step 2		
Gr. 2 arithmetic fluency	—	—	—	–1.38***	.18	0.25	–0.86***	.21	.42	–0.70**	.22	.49
							Step 3			Step 3		
Gr. 4 arithmetic fluency	—	—	—	—	—	—	–1.09***	.19	.33	–0.60**	.22	.55
										Step 4		
Gr. 6 arithmetic fluency	—	—	—	—	—	—	—	—	—	1.17***	.24	.31
	Nagelkerke *R*^2^ = .22***			Nagelkerke *R*^2^ = .35***			Nagelkerke *R*^2^ = .43***			Nagelkerke *R*^2^ = .48***		
AUC	.80***^a^			.87***			.90***			.92***		

*Note.* Standardized scores of continuous predictors were used in the model to increase the comparability of the odds ratios related to different predictors. The beta-coefficients related to the final model obtained after all the steps in each model are reported in the table. LK = letter knowledge; RAN = rapid automatized naming; PA = phonological awareness.

a Area under the ROC curve calculated using only the significant predictors of MD status in adolescence.

* *p* < .05. ** *p* < .01. *** *p* < .001.

Subsequently, in Model 2, the significant predictors (FR_RD_, LK, and RAN) from Model 1 were included in Step 1, and Grade 2 reading fluency was added in Step 2. This change increased the portion of the explained variance from 18% to 36%, χ^2^(1) = 95.55, *p* < .001. In Model 2, RAN was no longer a significant predictor. Based on the AUC value (0.89), the model’s ability to predict RD statuses in adolescence was good. The number of correctly predicted individuals when using a cutoff probability that resulted in 89.7% sensitivity heightened to 69.8%, and the specificity of the model raised to 67.8%. Adding Grade 4 reading fluency (Model 3) into the previous model in Step 3 heightened the portion of the explained variance 11%, to 47%, χ^2^(1) = 55.14, *p* < .001. The predictability of the model was excellent (AUC = 0.91). In this model, the FR_RD_ was no longer significant, although it was close (*p* = .053). The number of correctly predicted individuals and the specificity of the model continued to increase at 78.1% and 76.9%, respectively. Grade 6 reading fluency (Model 4) in Step 4 increased the portion of the explained variance a further 5%, to 52%, χ^2^(1) = 30.23, *p* < .001. The predictability of the last model was excellent (AUC = 0.93). Family risk for reading difficulties (FR_RD_), LK, and all three reading fluency measures were significant predictors of adolescents’ RD status. Adding Grade 6 reading fluency no longer raised the number of correctly predicted individuals or the specificity of the model.

Next, we ran separate binary logistic regression analyses to examine the unique predictive values of reading fluency skills in Grades 2, 4, and 6 separately, because in schools, information regarding cognitive skills and parental measures are not necessarily available. It is important to understand whether information on the skill level is a sufficient predictor. The analyses revealed that identifying adolescents’ RD status was quite reliable each time (AUC = 0.85 for Grade 2 and 0.89 for Grades 4 and 6). Grade 2 reading fluency alone explained 31% of the outcome variance, χ^2^(1) = 144.72, *p* < .001. Respectively, Grade 4 and Grade 6 reading fluency each explained 40% and 41% of the outcome variance, χ^2^(1) = 189.03, *p* < .001, and, χ^2^(1) = 192.37, *p* < .001. However, the number of correctly predicted individuals and the specificity of the model were lower in each grade compared with the cumulative models, including FR_RD_, LK, and RAN.

As a final step, a supplementary post-hoc analysis based on gender differences between the RD and NoRD groups (see Table 1) was conducted to explore the potential predictive role of gender in relation to RD status. The inclusion of gender had varying effects on predictive models, leading to a greater explained variance ranging from 1% to 6% and increased model specificity from 3% to 15%. Notably, the most substantial enhancement was observed in the first model, including parental and preschool cognitive predictors. The portion of the explained variance raised from 18% to 24%, and specificity improved from 46.3% to 58.9%.

### Predicting MD Status in Adolescence

In arithmetic, FR_MD_ was more common, and parental education was lower in the MD group than in the NoMD group. The NoMD group outperformed the MD group in all cognitive and academic skills (see Table 2), and the effect sizes varied from moderate to large. Hierarchical binary logistic regression analysis revealed that neither FR nor parental education predicted adolescents’ MD status (see Table 5). In Step 2, counting and spatial relationships turned out to be the only significant predictors of adolescents’ MD status. The model explained 22% of the variance in adolescents’ MD status, and the model fit was barely good according to the AUC value (0.80). Using a cutoff probability that resulted in 89.3% sensitivity in binary logistic regression, the model correctly predicted adolescents’ MD status in 57% of cases due to low specificity (53.8%) (see Table 6).

**Table 5.** Classification Accuracy of Adolescent RD Status of Different Models at Cutoff Levels Resulting in 90% Sensitivity in Each Model.

Predictors	Probability level	Correct classification %	Sensitivity %	Specificity %
Parental and preschool cognitive predictors	.05	50.4	90.8	46.3
FR of RD, LK, RAN, and Grade 2 RF	.06	69.8	89.7	67.8
FR of RD, LK, RAN, and Grade 2 and 4 RF	.07	78.1	89.7	76.9
FR of RD, LK, RAN, and Grades 2, 4, and 6 RF	.06	78.0	89.4	76.8
Grade 2 RF	.05	62.2	89.7	59.4
Grade 4 RF	.06	73.3	89.7	71.7
Grade 6 RF	.05	70.3	89.4	68.4

*Note.* FR = family risk; RD = reading disability; LK = letter knowledge; RAN = rapid automatized naming; RF = reading fluency.

**Table 6.** Classification Accuracy of Adolescent MD Status of Different Models at Cutoff Levels Resulting in 90% Sensitivity in Each Model.

Predictors	Probability level	Correct classification %	Sensitivity %	Specificity %
Parental and preschool cognitive predictors	.05	57.0	89.3	53.8
CO, SR, and Grade 2 AF	.05	69.0	89.3	67.0
CO, SR, and Grades 2 and 4 AF	.07	77.0	89.3	75.8
CO, SR, and Grades 2, 4, and 6 AF	.08	81.8	89.0	81.1
Grade 2 AF	.05	60.6	90.5	57.6
Grade 4 AF	.06	72.2	90.5	70.4
Grade 6 AF	.06	71.8	90.2	70.1

*Note.* MD = mathematics difficulties; CO = counting skills; SR = spatial relations; AF = arithmetic fluency.

Next, adding Grade 2 arithmetic fluency into the previous model with significant kindergarten predictors (counting and spatial relationships) raised the portion of the explained variance from 22% to 35%, χ^2^(1) = 76.71, *p* < .001. The model’s ability to predict the MD status in adolescence was enhanced, with AUC values rising from 0.80 to 0.87. Both kindergarten measures remained significant after including Grade 2 arithmetic fluency in the model. The ability of the model to correctly predict individuals with MD in adolescence increased to 69% after adding Grade 2 arithmetic fluency to the model. Specificity increased to a level in which two-thirds of the affected children were correctly classified. Adding Grade 4 arithmetic fluency significantly increased the portion of explained variance of adolescents’ MD status by 8%, χ^2^(1) = 8.14, *p* = .004, total explained variance being 43%, and the model’s ability to predict adolescents’ MD status was excellent (AUC of 0.90). Counting and spatial relationships still made a unique contribution to predicting adolescents’ MD status. The number of correctly predicted individuals and the specificity of the model continued to increase at 77.0% and 75.8%, respectively. Finally, adding Grade 6 arithmetic fluency increased the portion of the explained variance by 5% up to 48%, χ^2^(1) = 28.17, *p* < .001. The predictability of the final model was excellent (AUC = 0.92). Counting and spatial relationships were no longer significant predictors, whereas arithmetic fluency in Grades 2, 4, and 6 were. Adding Grade 6 arithmetic fluency into the model continued to heighten the number of correctly identified individuals and specificity of the model being 81.8% and 81.1%, respectively.

Separate binary logistic regression analyses examining the unique predictive value of the arithmetic fluency skills of Grades 2, 4, and 6 revealed that the prediction of adolescents’ MD status was quite reliable each time, as the AUC was 0.85, 0.88, and 0.89 in Grades 2, 4, and 6, respectively. Grade 2 arithmetic fluency explained 31% of the outcome variance, χ^2^(1) = 142.70, *p* < .001. Grade 4 and Grade 6 arithmetic fluency each explained 35% and 39% of the outcome variance, respectively, χ^2^(1) = 164.58, *p* < .001, and, χ^2^(1) = 178.63, *p* < .001. Likewise, as for reading, the number of correctly predicted individuals and the specificity of the model were lower in each grade compared with the cumulative models, including preschool counting and spatial relationships.

## Discussion

The present study examined how early and with which constellation of measures we can predict adolescents’ dysfluency in reading and arithmetic. We included three types of potential predictors: parental measures (parental education and their own reading and mathematics difficulties), kindergarten-age cognitive skills (LK, PA, RAN, counting, number concept, and spatial relations), and school-age reading and arithmetic fluency measured at three preadolescence timepoints: right after the basic skill acquisition phase (Grade 2) and during the fluency-building phase (Grades 4 and 6). The main findings indicated that, first, adolescent difficulties could be moderately predicted by parental measures and kindergarten cognitive skills in reading, but only by cognitive skills in arithmetic. However, the specificity of the models remained low. Second, adding school-age fluency measures clearly increased the accuracy of the constructed models for predicting adolescent dysfluency in reading and arithmetic. Importantly, each phase of skill development possessed its own unique predictive value which suggests that although early dysfluency is a significant predictor of later dysfluency, individuals’ developmental paces vary, and the prediction improves across time. This finding means also that delay in achieving fluency in these academic skills does not necessarily always indicate persistent difficulties, and thus, in some cases only longer monitoring reveals the true difficulties including low response to instruction and support at school. Third, models that included kindergarten-age cognitive skills and familial risk for RD were more accurate than models with only school-age fluency measures. Therefore, it is crucial to highlight the significance of documenting information related to FR and to assess cognitive skills during the kindergarten years and use them in the predictive models. Utilizing this information in conjunction with monitoring skill development throughout the school years enhances the provision of timely preventive support. Finally, specific models were found for reading and arithmetic. Family risk was a significant predictor only in reading. Cognitive predictors differed, and the additive predictive effects of school-age fluency measures were clear only up to Grade 4 in reading. Meanwhile, the arithmetic fluency at Grade 6 continued to raise the number of correctly predicted individuals and the specificity of the model.

### Parental Variables and Kindergarten Skills as Predictors of Dysfluency

With a knowledge of FR and kindergarten cognitive skills, we could predict the reading dysfluency statuses in adolescents with moderate accuracy and arithmetic dysfluency status with good accuracy. When using high-sensitivity criteria (90%), less than half of adolescents (46%) were predicted correctly to have dysfluency in reading, while over half of adolescents (57%) were predicted correctly to have dysfluency in arithmetic. Interestingly, both in reading and arithmetic, the cumulative models including FR and kindergarten skills together with fluency in reading or arithmetic correctly predicted more individuals and had higher specificity values than any models, including only fluency in the corresponding domain at a certain grade. Moreover, in regression models, kindergarten predictors were found to have unique predictive effects beyond reading/arithmetic fluency in Grades 2 and 4, and even Grade 6.

Letter knowledge was found to be a unique predictor of dysfluency status in reading and remained important even after including Grade 6 reading fluency in the model. Rapid automatized naming was the other significant kindergarten cognitive indicator. However, its unique predictive effect disappeared after including Grade 2 reading fluency in the model. The discovery that knowledge of letters’ names (LK) remains a unique predictor of dysfluency in adolescence, even after accounting for reading fluency in elementary school, is intriguing. This suggests that a delay in learning letter names holds significant long-term predictive value. Consequently, this information should be considered when planning educational support in schools, and added to monitoring of reading skills. It is important to note, however, that the predictive effect of rapid serial naming of letters was not examined in this study. This is because automaticity in the retrieval process can only be assessed after letters have been formally taught. Thus, the role of rapid serial naming of letters should be explored among school-age children in future studies, along with reading fluency to complement present findings regarding the RAN.

Similarly, counting and visuospatial skills were unique predictors for dysfluency in arithmetic among adolescents and continued to predict dysfluency even after including arithmetic fluency in Grades 2 and 4 in the model. However, they did not remain significant after including arithmetic fluency in Grade 6. Thus, although the adolescents’ reading and arithmetic dysfluency groups performed lower in all kindergarten cognitive skills than the controls without dysfluency in that domain, only the LK and RAN on reading and counting and visuospatial skills on arithmetic had unique long-term predictive effects. The unique long-term predictive effect of kindergarteners’ cognitive skills on dysfluency in adolescence suggests that LK and verbal counting skills at the age of 6 years are much more than merely a proxy for basic reading and arithmetic skills.

Only a handful of previous studies have predicted the reading dysfluency status in adolescence using kindergarteners’ skills (Landerl & Wimmer, 2008; Torppa et al., 2015). Even fewer studies concern the long-term predictors of arithmetic dysfluency status or mathematics difficulties (Koponen et al., 2019). More specifically, Landerl and Wimmer (2008) found in a German-speaking sample that LK did not predict Grade 8 RD. Simultaneously, RAN assessed at the beginning of Grade 1 was a significant predictor, although it was added to the model after LK, nonverbal intelligence quotient (IQ), phonemic awareness, and phonemic short-term memory. Torppa et al. (2015) found that the strongest effect sizes indicating the mean-level difference between persistent dyslexia (Grades 2 and 8) and no-dyslexia groups were found in PA from age 6.5 onward, RAN, and LK. Similarly, in a recent study using the same data as in the present study (Psyridou et al., 2022), RAN assessed before school entry was found to be the most important predictor of dysfluency in reading when difficulties were defined in Grade 9. Their findings also provided evidence of dysfluency predicted by LK. However, none of these previous studies considered reading fluency in the early school grades simultaneously in the same model when examining the effect of RAN, as was the case in our study. In fact, in the present study, kindergarten-age RAN was a significant predictor of dysfluency status in adolescence before reading fluency in Grade 2 was added to the model. This finding is in line with the view regarding RAN as a microcosm of the processes involved in reading (Norton & Wolf, 2012).

Letter knowledge, in contrast, continued to predict persistent dysfluency statuses in lower secondary school even after including reading fluency in Grades 2, 4, and 6 in the models. Meanwhile, in a study by Landerl and Wimmer, the effect of LK was important in Grade 4 only. Letter knowledge has previously been demonstrated to be one of the key predictors of dyslexia in Finnish orthography among primary schoolchildren. Letter knowledge has previously been demonstrated to be one of the key predictors of dyslexia in Finnish orthography among primary schoolchildren (Puolakanaho et al., 2007) and persistent dyslexia across Grades 2 to 8 (Torppa et al., 2015) in a sample with a high prevalence of familial risk for dyslexia. In the present study, novel information was obtained by depicting LK as a strong early indicator of risk for dysfluency in reading. Letter knowledge provides predictive power over RAN and reading fluency in elementary school.

Our finding of counting as a unique long-term predictor of MD beyond arithmetic fluency assessed in Grades 2 and 4 corroborates the findings of Koponen et al. (2019), indicating that almost all students facing difficulties in Grade 7 mathematics exhibited difficulties in counting in kindergarten. Moreover, their analyses with continuous variables demonstrated the predictive effect of counting on Grade 7 mathematics performance above Grade 4 arithmetic skills. The limitations in that study were the small original sample size and, thus, the subsequently small number of children with MD (*N* = 200 and 13 children with MD). In addition, they were identified to have an MD based on information from only one time point (Grade 7). The present study also extended the previous findings by revealing that counting had a unique predictive effect, even when analyzed concurrently with other numeric, language, and visual cognitive skills, and predicted persistent dysfluency in arithmetic among adolescents for whom data were available for both Grades 7 and 9. However, together the findings of these two studies highlight that counting is not only a tool for early arithmetic calculation relying typically strongly on the use of counting-based (procedural) strategies, but it is also a unique predictor of dysfluency in adolescence beyond arithmetical skills in Grades 2 and 4. It is well justified to assert that, given the strong predictive value of counting measured before school entry, counting skills should play a larger role in future studies aimed at unraveling individual pathways to mathematical difficulties, especially arithmetic dysfluency.

The analyses of the present study indicated that visuospatial cognitive skills were an additional risk indicator of dysfluency in arithmetic. Several studies have depicted an association between visuospatial skills and arithmetic (Kyttälä & Lehto, 2008). However, the present study illustrated for the first time that visuospatial skills have a unique long-term predictive effect on arithmetic dysfluency status among adolescents, even after including arithmetic fluency at Grades 2 and 4 in the analysis. The relationship between visuospatial skills and arithmetic (or mathematics in general) is unclear. Several possible accounts have been suggested, such as the spatial representation of numbers (e.g., mental number line), shared neural processing, for example, the intraparietal sulcus (IPS), spatial modeling, or working memory (Hawes & Ansari, 2020). The assessment of spatial visualization in the current study involves a task requiring the multistep processing of spatial information, containing the skill to analyze a collection of shapes and adeptly integrate them to conceptualize a novel design. The interrelation between pure spatial visualization and, conversely, the part–whole knowledge required in mathematics, as well as the potential significance of spatial visualization in multidigit calculation and the comprehension of operations with rational numbers, warrants in-depth investigation in future research.

### Primary School Skills as Predictors of Dysfluency

As expected, after including children’s fluency levels in reading or arithmetic in Grade 2, the predictability of the model heightened and was considered good regarding reading and arithmetic dysfluency status. Thus, adding information on children’s earlier fluency levels in reading and arithmetic after 2 years of formal instruction and practice clearly sharpened the accuracy of predicting dysfluency in adolescence. This can partly attest to the stability of fluency measures across age. The finding is also important for gaining additional understanding of the development of the skills in question. It suggests that while reading- and arithmetic-related cognitive skills form an important foundation for later learning or reading and arithmetic, they do not solely determine children’s responses to educational instruction and training and the path along which their academic skills develop during their primary school years. Rather, indicators reflecting the successful acquisition of basic techniques in arithmetic and reading skills during the initial grades of primary school clearly provide additive information above the kindergarten cognitive skills that can be considered pre-skills relevant for the academic skills taught at school.

Regarding both reading and arithmetic dysfluency status in adolescence, the inclusion of Grade 4 skills ameliorated the predictability of the models to excellent. Grade 2 children in the Finnish language context have typically acquired high accuracy in reading and arithmetic skills. Correspondingly, promoting fluency in these academic skills is highlighted as a key focus in reading and arithmetic instruction. A shift in educational instruction from a focus on securing accuracy in decoding or basic calculation toward a focus on supporting fluency is likely to add variance between children, which predicts dysfluency in adolescence in reading and arithmetic. For example, in arithmetic, multiplication tables have been introduced and intensively practiced after second grade. Compared with addition and subtraction, it is known that multiplication tables are practiced to a larger extent by drilling aiming to direct fact retrieval. Thus, Grades 3 and 4 are important time periods for arithmetic fluency development and bring new variance between individuals related to differences in fact retrieval skill while at Grade 2 differences in procedural calculation fluency might be more emphasized providing unique predictive information as well. This highlights the importance of continually monitoring the development of academic skills throughout primary school. Although the predictability of the models and the proportion of the explained variance continued to increase when Grade 6 fluency was included, the additive value was less clear compared with the increased predictivity from Grades 2 to 4.

When taken alone, fluency at a certain grade had fairly good but not excellent predictivity. The number of correctly predicted individuals and the specificity of the model were less in each grade analyzed separately than when examining the cumulative models, including children’s kindergarten-age skills and parental variables. This finding suggests that kindergarten-age skills and parents’ difficulties in reading can be important indicators of risks for learning difficulties based on neurocognitive deficits. However, there can be other reasons for low performance at certain assessment points, such as motivational or environmental factors, which can have a more situational basis and more transient effects on skill development. These factors and contextual changes can partially explain why previous academic fluency was not a perfect predictor. Hence, a more comprehensive approach is needed, rather than focusing solely on academic fluency at specific time points.

### Comparing Predictions of Dysfluency in Reading and Arithmetic

Although the unique predictive effects of fluency in Grades 4 and 6 were rather similar for both reading and arithmetic dysfluency status in adolescence, only arithmetic fluency in Grade 6 continued to increase the number of correctly predicted individuals with dysfluency in adolescence and the specificity of the model, which was not the case for reading. Differences between the two academic domains, influencing their operationalizations, might partially explain this finding. Basic skills (covering the four arithmetic operations in mathematics and decoding in reading) were taught and practiced intensively during the first two school years in both domains. Mathematics instruction, however, follows a series of successive shifts toward more complex skill accumulation demands reflecting the hierarchical nature of the subject matter: from addition and subtraction to multiplication and division, from single-digit to multidigit number operations, and from natural numbers to rational numbers. Thus, in learning arithmetic, continuous requirements prevail for learning new knowledge in addition to mastering basic arithmetic calculations, and this was taken into account when measuring arithmetic fluency. This could also explain our findings, which indicated that reading fluency observed across primary school grades exhibited a slightly stronger predictive effect on dysfluency status in adolescence compared with the predictive impact of arithmetic fluency. In contrast, a slightly stronger predictive effect of kindergarten variables was found for dysfluency status in arithmetic rather than in reading. Given that arithmetic fluency among adolescents encompasses a broad range of subskills, including the knowledge of place values for multidigit numbers, the base-ten system, and rational numbers, it is reasonable that it relies not only on basic numeric skills, but also on non-numeric cognitive domains, such as visuospatial skills.

The present study also documented distinct predictive factors for adolescent dysfluency status in reading and arithmetic. Regarding the role of parental factors, FR_RD_ was the only predictor of RD dysfluency status in adolescence. Its unique predictive value exceeded kindergarten-age cognitive skills, even after including Grade 6 reading fluency. Family studies and twin studies have provided robust evidence that reading and mathematics difficulties have a heritable component. However, alongside genetics, environmental factors also help manifest learning difficulties. To delineate the distinct roles of each factor in familial risk, genetically sensitive research designs should be pursued in future investigations (Hart et al., 2021). None of the parental predictors predicted dysfluency status in arithmetic. Differences in findings related to the long-term predictive value of FR_RD_ and FR_MD_ might be related to the nature of the skill. Mathematical skills cover a wider set of subskills than reading. Hence, difficulties might also reveal greater heterogeneity in mathematics. Although dysfluency in arithmetic is a central feature in MD, it is not the only one. For instance, some individuals may struggle more than others with number system knowledge, such as understanding place values and magnitudes of larger numbers or decimals (e.g., Geary, 1993). The question related to mathematics difficulties in families was not specified to cover only arithmetic dysfluency. In the future, it would be advisable to inquire about more specific information on the nature of the difficulties to obtain a clearer picture of FR_MD_ as a predictor of children’s difficulties in mathematics.

### Practical Implications

Understanding the cognitive building blocks and their hierarchy provides knowledge of the skills that should be monitored before school entry for the early prediction of children who need support, with the goal of preventing or attenuating later difficulties in these two important academic skills. Information guiding and augmenting early identification are meaningful for teachers at schools and kindergartens when allocating educational support resources, as resources for intensified special educational support are traditionally delivered more in a categorical than dimensional fashion (i.e., special educational support is not equally distributed, and resources are limited and not available to all). Assessments of both LK and counting skills can be easily administered. After further validation, they have the potential to be included in short screening tests and protocols used to evaluate school readiness. The earlier that schools and early childhood units can reliably predict children at risk of developing learning difficulties, the earlier that targeted support can be provided.

Empirical evidence exists regarding effective methods for acquiring LK (e.g., Verhoeven et al., 2020) and early number skills, including counting (Nelson & McMaster, 2019). Nevertheless, the extent to which these foundational skills—and remedial support in attaining a sufficient level in them—facilitate the progression of reading and arithmetic, while potentially mitigating or averting difficulties, remains uncertain. It is also possible that the ability to acquire LK and counting reflects key neurocognitive processes integral not only for these foundational skills but also the subsequent development of proficient reading and arithmetic abilities. Longitudinal and individualized support for learning beyond the early preventive support phase is likely needed for a substantial subgroup of students showing problems in learning these pre-skills.

Notably, when setting the desired sensitivity criteria at a high level (e.g., the criterion that 90% of those with MD in adolescence will be predicted), the specificity level reached with kindergarten predictors together with parental predictors is rather low. In reading, little less than half and in arithmetic little more than half of adolescents without difficulties were correctly predicted. There is, therefore, an evident need for monitoring skill development at regular intervals with reliable assessment tools, both in arithmetic and reading skills, to receive good specificity.

### Limitations

The present study has limitations to consider when making interpretations or generalizations. First, learning difficulty status was defined using a cut-off criterion, which was set at the lowest 16th percentile in both Grades 7 and 9. The setting of a cut-off is always somewhat arbitrary, and categorizing continuous variables has known limitations, including the effects of measurement error, which can decrease the reliability of identification (Branum-Martin et al., 2013). However, using the double-occurrence criterion (i.e., the occurrence of difficulties across lower secondary school) was required for the present study, which is likely to increase the possibility of identifying adolescents with true difficulties. Previous studies have rarely used the double-occurrence criterion when examining predictors of learning difficulty, although the persistent nature of difficulties is included in the diagnostic criteria for learning disabilities (e.g., *Diagnostic and Statistical Manual of Mental Disorders* [5th ed.; *DSM-5*; American Psychiatric Association, 2013]).

The second limitation relates to the selection of predictors, as not all relevant numeric predictors could be included. For example, magnitude comparison, especially symbolic number comparison, has been suggested as one of the core predictors of mathematics skills and MD (e.g., Schneider et al., 2017) and should be included in future studies. Third, conducting individual assessments of reading fluency through read-aloud tasks would have been preferable. Unfortunately, the resources available for this large-scale longitudinal study did not permit individual assessments of all participants. Therefore, we had to resort to group assessment tasks instead. Fourth, FR was measured using self-reports with a single question. In addition to questionnaires, parents’ skill assessments should be considered in future designs to provide a more accurate evaluation of a family’s risk of learning difficulties.

Fifth, during the formal schooling period, students identified as being at risk typically receive some form of specialized education services or interventions at some point of their educational career. Regrettably, we did not have this information available to consider in our analysis. Sixth, considering the potential differences between the family sample in Finland and family samples in other countries, such as the United States, it is crucial to acknowledge this as a potential limitation in our current research. In Finland, families are relatively homogeneous in terms of socioeconomic, ethical, cultural, and linguistic factors. Consequently, the role of family variables may diverge from countries with a larger variability in socioeconomic factors, for instance. Conducting comparative studies across countries could offer valuable insights into the impact of sociocultural factors on family variables, their implications for learning academic skills, and the manifestation and stability of learning difficulties among older students.

## Conclusion

The main findings of this work indicate that FR_RD_ and cognitive skills at kindergarten age, which are acknowledged pre-skills associated with the early acquisition of key academic skills, are potential long-term predictors of dysfluency in reading and arithmetic in adolescence. Consequently, their role as key components of an early screening tool should be examined in the future. In practice, this would mean defining and exploring different cut-offs for each measure, identifying children at risk based on scores below cut-off in each measure, and exploring what kind of combination of risk indices works best/receives strongest evidence for reliable early identification of adolescent’s dysfluency. The level of reading and arithmetic fluency after the first 2 years of schooling and later in Grades 4 and 6 further specified the prediction of dysfluency problems in adolescence, highlighting the need to monitor skill development during the primary school years. However, despite the excellent predictivity of the final models, notably, even after 6 years of formal instruction and practice, developmental changes take place in two basic academic skills. Although the skill levels in Grades 4 and 6 strongly predict the dysfluency status in reading and arithmetic, they did not determine the paths in reading and arithmetic. Further studies are needed to specify the developmental mechanisms, including interactions between the individual and the environment, which can enhance resilience and resolve difficulties despite the early risk of learning difficulties.

## Supplemental Material

sj-docx-1-ldx-10.1177_00222194241275644 – Supplemental material for Predicting Adolescent Arithmetic and Reading Dysfluency Supplemental material, sj-docx-1-ldx-10.1177_00222194241275644 for Predicting Adolescent Arithmetic and Reading Dysfluency by Tuire Koponen, Kenneth Eklund, Kaisa Aunola, Anna-Maija Poikkeus, Marja-Kristiina Lerkkanen and Minna Torppa in Journal of Learning Disabilities

sj-docx-2-ldx-10.1177_00222194241275644 – Supplemental material for Predicting Adolescent Arithmetic and Reading Dysfluency Supplemental material, sj-docx-2-ldx-10.1177_00222194241275644 for Predicting Adolescent Arithmetic and Reading Dysfluency by Tuire Koponen, Kenneth Eklund, Kaisa Aunola, Anna-Maija Poikkeus, Marja-Kristiina Lerkkanen and Minna Torppa in Journal of Learning Disabilities
