# Supplementary material for: Predicting Adolescent Arithmetic and Reading Dysfluency
Source: J Learn Disabil. 2024 Sep 24;58(3):163–78. doi: 10.1177/00222194241275644 (PMC11993818; doi:10.1177/00222194241275644)
Supplement: sj-docx-1-ldx-10.1177_00222194241275644 – Supplemental material for Predicting Adolescent Arithmetic and Reading Dysfluency [file sj-docx-1-ldx-10.1177_00222194241275644.docx]

| Measure | Task | Year | Description | Scoring | Reliability |
| --- | --- | --- | --- | --- | --- |
| Parental Measures | Parental education |  | Mothers and fathers were asked to indicate their own education level on a 7-point scale: 1 = no vocational education, 2 = vocational courses (4 months), 3 = vocational school degree, 4 = vocational college degree, 5 = polytechnic degree or bachelor’s degree, 6 = master’s degree, and 7 = licentiate or doctoral degree. | The sum score was computed as an average of both parents’ individual scores. |  |
| Kindergarten Measures | Family risk for learning difficulties |  | Parents were asked to indicate on a 3-point scale whether they had clear difficulties, some difficulties, or no difficulties in reading or math. | 1 was coded, if the mother or the father reported that she or he had experienced some or clear learning difficulties, otherwise 0 |  |
|  | Initial phoneme identification | Fall 2006 | Each child was shown four pictures of objects, which were named by the experimenter. The child was then asked to select the correct picture on the basis of the oral presentation of the initial phoneme relating to one target (e.g., “At the beginning of which word do you hear ____?”) | One point was given for every correct answer. Max 10. | Cronbach’s alpha= .75 |
|  | Letter knowledge | Fall 2006 | Letter knowledge was assessed with a list of the 29 letters of the Scandinavian variant of the Latin alphabet used in Finnish. The child had to name aloud the letter. Either a phoneme or letter name was regarded as correct. | One point was given for every correct answer. response. Max 29. | Cronbach’s alpha= .95 |

|  | RAN | Spring 2007 | The rapid naming of objects was assessed using the standard procedure (see Denckla & Rudel, 1974), in which the child was asked to name as rapidly as possible a series of visual stimuli with which they had become familiar. Matrices of 50 items (five stimuli 10 times) were used. The child’s performance was timed, and errors and self-corrections were documented. | Total matrix completion time in seconds. | - |
| --- | --- | --- | --- | --- | --- |
|  |  |  |  |  |  |
|  | Counting | Spring 2007 | Forward and backward counting were assessed using the following four items: counting forward from number 1 (counting was stopped after 31), counting forward from number 6 to 13, counting backward from number 12 (counting was stopped after 7), and counting backward from number 23 to 1 | 2 points were awarded for the correct outcome, 1 point for completing the task with up to two errors, and 0 points if the child made more than two errors or failed to complete the task Max 8. | Cronbach’s alpha= .63 |
|  | Number concepts | Spring 2007 | Number concept skill is a combined measure of ordinal and cardinal number knowledge as well as knowledge of basic mathematical concepts. The child saw a number and was asked to draw corresponding amount of balls or alternatively saw balls and was asked to select corresponding number from the five choices. Child was asked to draw balls according to instructions “*as many*”, “*one more*”, “*one less*” and mark the “*first*”, “*fourth*” and “*seventh*” ball. | One point was given for every correct answer. Max 9. | Cronbach’s alpha= .62 |
|  | Spatial relations | Spring 2007 | Visuo-spatial skills were assessed using a subtest of spatial relations from the Woodcock and Johnson ([1977](https://srcd.onlinelibrary.wiley.com/doi/full/10.1111/cdev.12173#cdev12173-bib-0045)) test battery. Child is required to identify the subset of pieces needed to form a complete shape with multiple-point scored items (i.e., *“Two of these pieces go together to make this. Tell me which two pieces.*”). It involves complicated, multistep manipulations of spatial information (i.e., detecting multiple spatial forms or shapes, rotating or manipulating them in the imagination, and matching). The test included 31 items in order of increasing difficulty and one point was given for each correct item within the time-limit of 3 minutes | One point was given for every correct answer. Max 31. |  |
