# Supplementary material for: Predicting Adolescent Arithmetic and Reading Dysfluency
Source: J Learn Disabil. 2024 Sep 24;58(3):163–78. doi: 10.1177/00222194241275644 (PMC11993818; doi:10.1177/00222194241275644)
Supplement: sj-docx-2-ldx-10.1177_00222194241275644 – Supplemental material for Predicting Adolescent Arithmetic and Reading Dysfluency [file sj-docx-2-ldx-10.1177_00222194241275644.docx]

JOURNAL OF LEARNING DISABILITIES SUPPLEMENTAL FILE

Table S2. Spearman Correlations of Family Risk of RD and MD, Parental education, Pre-school Cognitive predictors, and Reading and Arithmetic Fluency in Grade 2, 4 and 6, and Adolescent RD and MD status.

ARTICLE TITLE: Predicting Adolescent Arithmetic and Reading Dysfluency

|  |  | 2. | 3. | 4. | 5. | 6. | 7. | 8. | 9. | 10. | 11. | 12. | 13. | 14. | 15 | 16. | 17. |
| --- | --- | --- | --- | --- | --- | --- | --- | --- | --- | --- | --- | --- | --- | --- | --- | --- | --- |
| 1. | FR of RD | .38*** | -.09** | -.10** | -.14*** | .05 | -.10** | -.05 | -.03 | -.11** | -.08* | -.11** | -.07* | -.07* | -.07* | .12*** | .03 |
| 2. | FR of MD | - | -.13*** | -.04 | -.04 | .03 | -.02 | -.01 | -.01 | -.05 | -.07* | -.09 | -.05 | -.07* | -.04 | .09** | .07* |
| 3. | Parent’s education |  | - | .22*** | .19*** | -.14*** | .18*** | .13*** | .14*** | .13*** | .15*** | .12*** | .16*** | .20*** | .21*** | -.09** | -.10** |
| 4. | Letter Knowledge |  |  | - | .60*** | -.33*** | .56*** | .21*** | .20*** | .36*** | .36*** | .31*** | .31*** | .30*** | .30*** | -.24*** | -.22*** |
| 5. | Phonological awareness |  |  |  | - | -.26*** | .40*** | .19*** | .17*** | .30*** | .26*** | .23*** | .24*** | .20*** | .24*** | -.16*** | -.15*** |
| 6. | RAN |  |  |  |  | - | -.26*** | -.21*** | -.13*** | -.33*** | -.34*** | -.35*** | -.29*** | -.27*** | -.27*** | .22*** | .15** |
| 7. | Counting |  |  |  |  |  | - | .24*** | .20*** | .37*** | .36*** | .32*** | .47*** | .47*** | .40*** | -.19*** | -.27*** |
| 8. | Spatial relations |  |  |  |  |  |  | - | .12*** | .24*** | .27*** | .27*** | .24*** | .29*** | .29*** | -.12*** | -.20*** |
| 9. | Number concept |  |  |  |  |  |  |  | - | .22*** | .20*** | .16*** | .19*** | .20*** | .21*** | -.08* | -.11*** |
| 10. | Gr. 2 reading fluency |  |  |  |  |  |  |  |  | - | .65*** | .60*** | .43*** | .40*** | .39*** | -.35*** | -.21*** |
| 11. | Gr. 4 reading fluency |  |  |  |  |  |  |  |  |  | - | .69*** | .43*** | .45*** | .36*** | -.39*** | -.18*** |
| 12. | Gr. 6 reading fluency |  |  |  |  |  |  |  |  |  |  | - | .42*** | .43*** | .42*** | -.48*** | -.22*** |
| 13. | Gr. 2 arithmetic fluency |  |  |  |  |  |  |  |  |  |  |  | - | .70*** | .61*** | -.20*** | -.35*** |
| 14. | Gr. 4 arithmetic fluency |  |  |  |  |  |  |  |  |  |  |  |  | - | .69*** | -.22*** | -.38*** |
| 15. | Gr. 6 arithmetic fluency |  |  |  |  |  |  |  |  |  |  |  |  |  | - | -.22*** | -.40*** |
| 16. | RD status |  |  |  |  |  |  |  |  |  |  |  |  |  |  | - | .21*** |
| 17. | MD status |  |  |  |  |  |  |  |  |  |  |  |  |  |  |  | - |

*Note.* * *p* < .05, ** *p* < .01, *** *p* < .001. FR= family risk; RD= reading difficulties; MD= math difficulties; NoRD/NoMD= not difficulties in corresponding domain
